# Parent–Child Relationships, Parental Control, and Adolescent Mental Health: An Empirical Study Based on CEPS 2013–2014 Survey Data

**DOI:** 10.3390/bs15010052

**Published:** 2025-01-08

**Authors:** Tao Xu, Jiyan Ren

**Affiliations:** Department of Social Work, College of International Education and Social Development, Zhejiang Normal University, Jinhua 321004, China

**Keywords:** adolescent mental health, parent–child relationship, parental control, moderating effects

## Abstract

Previous research has consistently showed a close relationship between the quality of parent–child relationships and adolescents’ mental health. However, the relationship between parental control and adolescents’ mental health has remained controversial. This study utilized baseline data from the China Education Panel Survey (CEPS 2013–2014) to analyze the impact of parent–child relationships on adolescents’ mental health. The results indicated that parent–child relationships significantly influenced adolescents’ mental health. Parental control moderated the impact of parent–child relationships on adolescents’ mental health: When the parent–child relationship was good, appropriate behavioral control by parents strengthened the positive effect of a good parent–child relationship on adolescent mental health. Conversely, when the parent–child relationship was poor, parental control intensified the negative impact of a poor parent–child relationship on adolescent mental health. Furthermore, heterogeneity analysis revealed gender differences in the moderating effect of the parent–child relationship: compared to boys, the moderating effect of parental control on the parent–child relationship was more significant among girls.

## 1. Introduction

The parent–child relationship is a fundamental aspect of human development, significantly influencing various dimensions of a child’s life. This relationship, characterized by emotional bonds, communication patterns, and behavioral dynamics, serves as the foundation upon which children build their understanding of the world and their place within it. Among the numerous facets of parenting, the degree of parental control exercised has been a focal point of extensive psychological research. Parental control, encompassing both behavioral regulation and psychological autonomy, plays a crucial role in shaping adolescent mental health (Steinberg, 2019; Smetana et al., 2015).

Adolescence is a critical period marked by rapid physical, emotional, and social changes. During this transitional stage, the quality of parent–child interactions can have profound implications for adolescents’ mental well-being. Positive relationships, characterized by warmth, support, and appropriate autonomy granting, are often associated with better psychological outcomes (Laursen & Collins, 2009; Pinquart, 2016). Conversely, excessive or insufficient parental control can contribute to various psychological issues, including anxiety, depression, and behavioral problems (Barber & Xia, 2013; Laird et al., 2010).

Recent studies have further underscored the importance of balanced parental control. For instance, Barber et al. finds that adolescents with parents who maintain a balanced approach to control and autonomy exhibit lower levels of anxiety and depression (Barber et al., 1994). Similarly, a longitudinal study by Steinberg highlights that authoritative parenting, which combines high responsiveness with appropriate control, is linked to higher academic performance and better mental health in adolescents (Steinberg, 2001).

Understanding the nuances of parental control and its impact on adolescent mental health is essential for developing effective parenting strategies and interventions. This article aims to explore the intricate relationship between parent–child interactions, the spectrum of parental control, and the effects on adolescents’ mental health. By synthesizing current research and theoretical perspectives, we seek to provide a comprehensive overview of how different parenting styles and practices influence adolescent psychological development and well-being.

### 1.1. Parent–Child Relationship and Adolescent Mental Health

Positive parent–child relationships, characterized by adolescents valuing parental opinions, maintaining open communication with parents, and perceived parental care and support, significantly enhance adolescents’ positive behavioral outcomes and emotional health (Ackard et al., 2006; Hair et al., 2008). Such relationships consistently reduce the risk of adolescent depression (Kandel & Davies, 1982; Mak et al., 2021). Close parent–child relationships can enhance adolescents’ self-esteem and physical satisfaction (Boutelle et al., 2009), facilitate the development of close peer relationships (Mayseless et al., 1998), improve psychological resilience (X. Wang et al., 2022), and mitigate the onset of mental health problems, thereby playing a crucial role in the holistic development of adolescents’ physical and mental health.

In contrast, negative parent–child relationships, characterized by conflicts and detachment, can lead to higher levels of depression (Pearson & Wilkinson, 2013). Relationships marked by high conflict levels can trigger more psychological and behavioral problems in early adolescents (McKinney & Renk, 2011). Beyond emotional attributes like intimacy and conflict, parent–child relationships also exhibit behavioral attributes, often defined as parental involvement. Parental involvement symbolizes familial cohesion and parental interest in children (Gerard et al., 2006) and significantly benefits adolescent mental health (Flouri & Buchanan, 2003). It influences adolescents’ subjective well-being by enhancing their self-efficacy (Yap & Baharudin, 2015; Yu & Ho, 2018), enabling them to perceive parental support (Flouri & Buchanan, 2003). A large-scale study across five countries shows that parental involvement helps adolescents cope with stress, reducing feelings of helplessness, loneliness, and anxiety, thereby safeguarding their healthy development (Pengpid & Peltzer, 2018).

Moreover, parent–child relationships exert a moderating effect on adolescent mental health by interacting with peer pressure. High-quality parent–child relationships enhance adolescents’ emotional functioning and adaptive capacity, reducing negative emotions when facing peer pressure. Conversely, low-quality parent–child relationships exacerbate adolescent depression caused by peer pressure (Hazel et al., 2014; Liu & Merritt, 2018).

### 1.2. Parental Control and Adolescent Development

Parental control is a common parenting practice that Barber distinguishes into two types: psychological control and behavioral control, each impacting adolescent development differently (Barber, 1996). Psychological control, which involves manipulating a child’s psychological and emotional development, is a negative form of control that infringes on the child’s self-awareness and is significantly associated with adolescent depression and problem behaviors. In contrast, behavioral control, which focuses on managing and guiding a child’s behavior, can reduce the occurrence of adolescent problem behaviors by providing necessary behavioral guidance (Smetana & Daddis, 2002). Researchers suggest that appropriate parental control is beneficial as it includes supervision and discipline, which provide children with guidance, convey standards of competence, and offer feedback on progress, thereby supporting the development of personal competence (Grolnick & Pomerantz, 2009; Skinner et al., 2005).

However, as adolescents age, their perception of parental authority changes. They often begin to protest against parental regulation in certain areas of their lives and seek autonomy in personal domains, which is crucial for their mental health development (Pomerantz & Wang, 2009; Smetana et al., 2005). Parental control is sometimes perceived by adolescents as control over personal domains, leading them to feel psychologically controlled rather than guided or protected. This perception can result in negative outcomes such as psychological anxiety (Darling et al., 2008; Kakihara & Tilton-Weaver, 2009; Smetana et al., 2002). Parental control over adolescents’ friendships, attire, and daily behaviors may cause emotional distress (Gerard et al., 2006). Maternal control, in particular, can lead to perfectionist tendencies and increase the likelihood of depression (Kenney-Benson & Pomerantz, 2005), and high levels of behavioral control reported by mothers are significantly associated with lower emotional regulation abilities in college students (Manzeske & Stright, 2009). Research by Luthar and colleagues indicates that a lack of parental supervision and poor parent–child relationships can increase the risk of anxiety and depression among adolescents from higher socioeconomic backgrounds (Luthar, 2003; Luthar & Becker, 2002).

Interestingly, parental control can also promote adolescent mental health. A comparative study of Chinese and American adolescent samples shows that parental control improves academic performance, and in American adolescents, it has a significant positive impact on emotional development (Q. Wang et al., 2007). Other studies indicate that low levels of parental behavioral supervision are significant negative predictors of children’s life satisfaction (Leto et al., 2019) and are associated with poorer mental health (Arat & Wong, 2016). A cross-sectional study supports the positive impact of high levels of behavioral control on adolescent mental health, showing a significant association between maternal behavioral control and lower levels of adolescent anxiety (Salaam & Mounts, 2016).

Despite the ambiguous impact of parental control on adolescent mental health (Rothenberg et al., 2020), research indicates that parental control significantly affects the parent–child relationship, although the specific direction of this impact is debated. Sorkhabi and Middaugh suggest that reasonable and firm parental control is associated with positive parent–child relationships, typically accompanied by low levels of parent–child conflict, greater adolescent self-disclosure, and more parental knowledge of adolescents’ lives (Sorkhabi & Middaugh, 2014). However, Bi and colleagues argue that parental disciplinary behavior often brings some frequency of parent–child conflict, especially when parents impose overly strict constraints and discipline, leading adolescents to desire behavioral autonomy and thus causing parent–child conflict (Bi et al., 2018).

The impact of parental control on adolescent health is significantly influenced by cultural context. Different cultures have distinct perceptions of parent–child relationships, individual autonomy, and power hierarchies (Hofstede, 1984; Triandis, 2018). These differences directly influence parenting styles. For instance, Chao finds significant disparities between the authoritarian parenting style common in Asian cultures and the authoritative style prevalent in Western cultures (Chao, 1994). In collectivistic cultures, parents often emphasize obedience to authority and collective honor, exerting stricter control over their children. This may, to some extent, limit adolescents’ autonomy and exploratory behaviors. Triandis demonstrates that in collectivist cultures, individual identity is closely intertwined with group identity, leading parents to prioritize collective interests in their parenting (Triandis, 2018). Conversely, in individualistic cultures, parents place greater emphasis on fostering their children’s independence, exercising less control. This may be more conducive to adolescents’ psychosocial well-being. Markus and Kitayama argue that individualistic cultures prioritize individual independence and self-realization, leading parents to cultivate autonomy and self-confidence in their children (Markus & Kitayama, 2014).

### 1.3. Analytical Framework and Research Hypotheses

**Figure 1:**
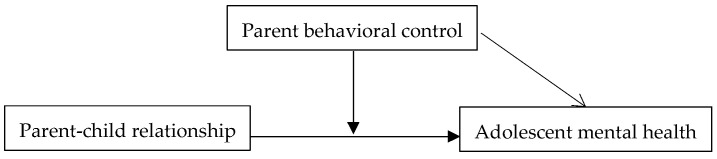
Analytical framework.

The controversy in previous research on the relationship between parental control and adolescent mental health primarily arises because the impact of parental control behaviors may differ under various contexts. In other words, parental control likely does not directly affect adolescent mental health but does so indirectly, potentially influenced by the quality of the parent–child relationship. Therefore, we have constructed a theoretical analysis model (see Figure 1). In this model, we first explore the direct effect of the parent–child relationship on adolescent mental health. Then, we examine how parental control, under different parent–child relationship contexts, indirectly moderates the impact of the parent–child relationship on adolescent mental health.

The impact of the parent–child relationship on mental health varies with different levels of parental control. Previous studies have consistently shown that a positive parent–child relationship contributes to adolescents’ mental health, while poor parent–child relationships are often associated with adolescent mental health problems (Mayseless et al., 1998; McKinney & Renk, 2011). Similarly, previous research has indicated that parental control can sometimes reduce adolescent mental health problems (Grolnick & Pomerantz, 2009; Skinner et al., 2005), while at other times, it can lead to them (Luthar, 2003; Manzeske & Stright, 2009). The relationship between parental control and parent–child relationship varies depending on the circumstances (Rothenberg et al., 2020). Therefore, when we theoretically explore the relationships among parental control, parent–child relationship, and adolescent mental health, if we consider parental control as a moderating variable, we can logically deduce that the effect of parental control on adolescent mental health may depend on the quality of the parent–child relationship. This study proposes that parental control may moderate the effect of the parent–child relationship on adolescent mental health. Specifically, there are two possible scenarios:

**Positive Promotion:** High levels of parental control may positively promote the beneficial effects of a good parent–child relationship. When the parent–child relationship is strong, high levels of parental control may make adolescents perceive more attention and guidance from their parents, fostering a close parent–child relationship and resulting in better mental health. Conversely, low levels of parental control in the context of a good parent–child relationship may make adolescents feel neglected, weakening the beneficial effects of a good relationship on mental health. Theoretically, the opposite could also be true, namely, that parental control might reduce the negative impact of a poor parent–child relationship on adolescent’ mental health problems.

**Negative Reinforcement:** High levels of parental control may exacerbate the detrimental effects of a poor parent–child relationship on adolescent mental health. When the parent–child relationship is weak, high levels of parental control may make adolescents feel more constrained and trigger parent–child conflicts, thereby reinforcing the negative impact of the poor relationship on adolescent mental health. Similarly, parental control may weaken the positive impact of a good parent–child relationship on adolescents’ mental health

Based on the above analysis, this paper proposes the following hypotheses:

**H1.**  *The better the parent–child relationship, the better the adolescent mental health*.

**H2a.**  *Parental control positively promotes the beneficial effects of a good parent–child relationship on adolescent mental health.*

**H2b.**  *Parental control negatively reduces the beneficial effects of a good parent–child relationship on adolescent mental health.*

**H3a.**  *Parental control reinforces the negative impact of a poor parent–child relationship on adolescent mental health.*

**H3b.**  *Parental control parental control may weaken the negative impact of a bad parent–child relationship on adolescents’ mental health.*

## 2. Methods

### 2.1. Data

This study utilizes data from the baseline China Education Panel Survey (CEPS) conducted during the 2013–2014 academic year. CEPS was a large-scale longitudinal survey project designed and implemented by the National Survey Research Center (NSRC) at Renmin University of China. The baseline survey took the average education level of the population and the proportion of the floating population as stratified variables. The specific methods of probability proportionate to size sampling (PPS), which included four stages of investigation, were used for the survey (T. Xu et al., 2024). The survey covered 28 county-level units, 112 schools, and 438 classes, surveying students in the first year and third year of junior high school, totaling 19,487 samples. To systematically investigate factors influencing adolescent mental health, this study focused on ninth-grade students. Data processing and analysis were performed using Stata 16.0.

### 2.2. Measurement

#### 2.2.1. Dependent Variable

Adolescent mental health issues: Measurement of adolescent mental health issues primarily utilizes a set of psychological test scales from the questionnaire based on PHQ-7 (Li et al., 2024; T. Xu et al., 2024), asking “In the past seven days, how often have you felt the following emotions?” The emotions assessed include depression, anxiety, unhappiness, meaninglessness of life, and sadness. Responses range from “never” to “always”, corresponding to scores from 1 to 5. Reliability tests indicate good consistency (Cronbach’s alpha = 0.8626). Mental health scores are calculated using the sum of scale scores, ranging from 5 to 25, where higher scores indicate poorer adolescent mental health. According to the scale, individuals with scores below 10 are classified as having excellent mental health. Those with scores between 10 and 20 are classified as having good mental health. Scores above 20 suggest the potential for significant mental health issues and warrant further professional evaluation.

#### 2.2.2. Independent Variables

Parent–child relationship: The core independent variable in analyzing adolescent mental health is the parent–child relationship. Due to the limitations of the measurement questions about parent–child relationships in the questionnaire, only these two subjective feeling questions are used to measure. Measuring parent–child relationships through subjective feelings is generally considered to be effective and is widely used (Cao & Liu, 2023; Liao et al., 2022). This study measures the perceived closeness between adolescents and their parents. Scores are derived from questions asking about relationships with both mother and father, with responses categorized as not close, average, or very close, scored from 1 to 3. The average score from both questions represents the parent–child relationship score, ranging from 1 to 3, where higher scores indicate a better relationship with parents. Generally speaking, a score of 1 indicates a poor relationship with parents, a score of 2 indicates neither good nor bad relationship, and a score of 3 indicates a very good relationship with parents.

#### 2.2.3. Moderating Variables

Parental control: This is measured using an 8-item scale assessing the strictness of parental control in various aspects such as school performance, friendships, and leisure activities. Responses are categorized as not strict, moderately strict, and very strict, scored from 1 to 3. Reliability testing shows good internal consistency (Cronbach’s alpha = 0.7769). The sum of scores from these items provides a comprehensive measure of parental control, ranging from 8 to 24, with higher scores indicating higher levels of parental control. Typically, scores below 8 indicate very weak parental control, while scores between 8 and 16 suggest moderate parental control. Scores above 16 may indicate very strict parental control.

#### 2.2.4. Control Variables

Control variables include adolescent age (continuous variable), gender (binary variable with female as the reference), household registration type (binary variable with rural household registration as the reference), family economic status (continuous variable where higher values indicate better economic conditions), and parental education level transformed into years of education (coded as 0 for no education, 6 for primary school, 9 for junior high school, 12 for vocational school/technical school, 15 for college associate degree, 16 for bachelor’s degree, and 19 for postgraduate education and above).

### 2.3. Research Strategy

This study employs ordinary least squares (OLS) regression to analyze adolescent mental health conditions. The model is expressed as follows:

Y = β_0_ + β_1_X + β_2_M + β_3_MX + e

where Y represents the score of adolescent mental health, X denotes the influence of the parent–child relationship on Y, and M represents the score of parental control, β_0_ is the constant term, and β_3_ represents the moderating effect. Through stepwise regression analysis of the parent–child relationship, parental control, and their interaction term, this study observes the impact of the parent–child relationship on adolescent mental health and the potential moderating effects of parental control.

## 3. Results

### 3.1. Descriptive Statistical Analysis

The descriptive statistics in Table 1 indicated that the overall level of mental health among adolescents was fairly good (M = 10.85; SE = 4.19), still with room for improvement compared to optimal levels. The average score for parent–child relationships (M = 2.60; SE = 0.48) generally reflected positive relationships. Meanwhile, the majority of adolescents perceived a moderate level of parental control (M = 18.41; SE = 3.19), though there were individual variations in perception. In terms of correlation analysis, parent–child relationships were significantly correlated with adolescent mental health (r = −0.243, *p* < 0.001), indicating that better parent–child relationships were associated with better mental health among adolescents. Additionally, parental control showed a significant negative correlation with adolescent mental health (r = −0.055, *p* < 0.001), suggesting that higher levels of parental control were associated with poorer mental health. Furthermore, a comparison of mean mental health scores between genders showed significant differences (*p* < 0.01), while differences among adolescents from different household registration types were not significant (*p* > 0.1). Regarding other demographic characteristics, age showed a significant positive correlation with adolescent mental health (r = 0.024, *p* < 0.05), suggesting that younger adolescents tended to have better mental health. Additionally, family economic status (r = −0.1, *p* < 0.001), father’s educational attainment (r = −0.021, *p* < 0.05), and mother’s educational attainment (r = −0.057, *p* < 0.001) all exhibited significant negative correlations with adolescent mental health problems. This indicated that higher family economic status and parental education levels contributed positively to adolescent mental health.

### 3.2. Regression Analysis

This study examined the relationships between parent–child relationship, parental control, and adolescent mental health and attempts to explore the moderating role of parental control. We first constructed Model 1 to investigate the direct impact of parent–child relationships on adolescent mental health, controlling for other factors. Secondly, we built Model 2 by introducing the parental control variable, and Model 3 was constructed to test the interaction between parental control and parent–child relationships on adolescent mental health. Finally, Models 4 and 5 were developed to test for gender differences. All multiple regression models underwent collinearity diagnostics prior to model building. Collinearity diagnostics showed all variables had variance inflation factors (VIFs) well below 10, indicating no issues with multi-collinearity.

In Table 2, results from Model 1 indicated a significant negative correlation between parent–child relationships and adolescent mental health (β = −2.074, *p* < 0.001). This meant the better the parent–child relationship, the fewer mental health problems the adolescent would have, and vice versa. This finding supported H1. Additionally, significant gender differences in adolescent mental health were observed, with adolescent girls exhibiting higher scores compared to boys. Moreover, poorer family economic status, non-rural household registration, lower maternal education levels, and higher paternal education levels were all significantly associated with poorer adolescent mental health.

**Figure 2:**
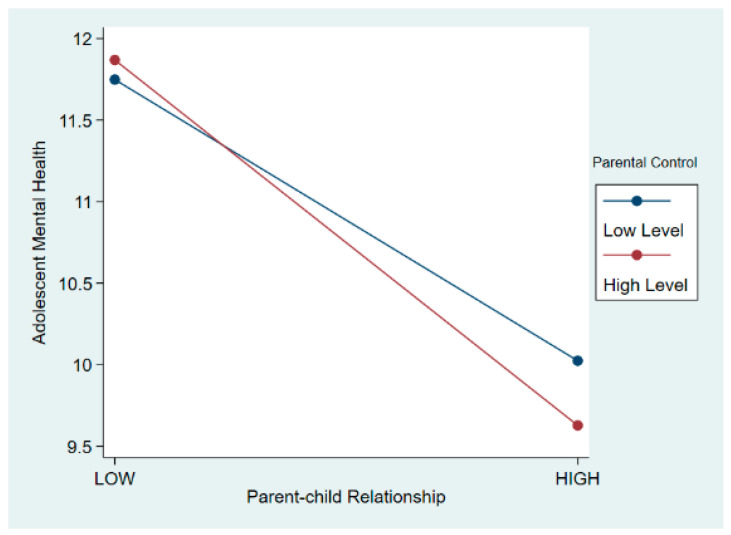
Moderating effect of parental control on the relationship between parental–child relationship and adolescent mental health.

In Model 2, after introducing the parental control variable, no significant correlation was found between parental control and adolescent mental health (β = −0.017, *p* > 0.1). Building on Model 2, Model 3 included an interaction term between parental control and parent–child relationships to observe the potential moderating effects of parental control. Results from Model 3 revealed that higher levels of parental control intensified the relationship between parent–child relationships and adolescent mental health (β = −0.087, *p* < 0.01). This implied that for adolescents perceiving better parent–child relationships, every one-unit increase in parental control reduced depressive scores by 2.197 units (−2.110–0.087). To visually depict the moderating effect pattern, a simple moderating effect plot was generated (see Figure 2). The plot illustrated that when parent–child relationships were strong, higher levels of parental control enhanced its inhibitory effect on adolescent mental health, indicating a positive moderating role of parental control (H2a supported, H2b unsupported). However, as depicted on the left side of the plot, higher levels of parental control exacerbated the mental health outcomes for adolescents with poor parent–child relationships, suggesting a negative moderating effect (H3a supported, H3b unsupported). Further simple slope *t*-test also confirmed these correlations (see S1 in Supplementary Materials).

### 3.3. Heterogeneity Analysis

Within the results of the aforementioned models, adolescent boys exhibited lower mental health scores. Existing research indicated that adolescent girls generally had significantly higher rates of depression compared to boys, with noticeable gender differences in adolescent mental health emerging around age 14. The likelihood of depressive episodes among adolescent girls increased sharply after puberty, with 15-year-old adolescent girls experiencing approximately twice the rate of depression compared to boys (Cyranowski et al., 2000; Thapar et al., 2012; Wade et al., 2002). Based on this, this study further explored differences in mental health scores among adolescents of different genders.

**Figure 3:**
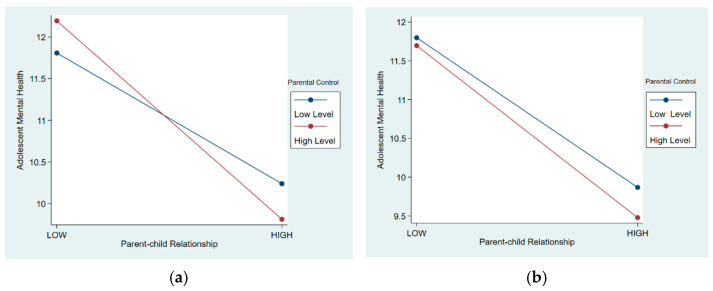
(**a**) Moderating effect for males. (**b**) Moderating effect for females.

Models 4 and 5 examined gender differences in the moderating effect of parental control on adolescent mental health. Controlling for other variables, the moderating effect of parental control showed significant impacts among adolescent girls (β = −0.138, *p* < 0.001), whereas it was not significant among adolescent boys (β = −0.0459, *p* > 0.1). Further comparison through simple effect analysis plots (see Figure 3a,b) visually illustrated that for boys, parental control did not significantly enhance the promotion of positive parent–child relationships. However, for girls, parental control significantly promoted the inhibitory effect of good parent–child relationships on mental health, while exerting a reverse moderating effect when parent control child relationships were poor.

### 3.4. Robustness Test

This study conducted robustness checks on the moderating effects of specific aspects of parental control (TV viewing time and return home time) by substituting them individually for overall parental control (see Table 3). The results of these checks indicated that parental control over TV viewing time showed a positive moderating effect on adolescent mental health (β = −0.391, *p* < 0.01), and similarly, parental control over return home time also demonstrated a positive moderating effect (β = −0.365, *p* < 0.01). These findings aligned closely with the moderating effect of overall parental control on adolescent mental health, suggesting robustness and consistency in the conclusions drawn.

## 4. Conclusions and Discussion

### 4.1. Discussion

Using the OLS regression method, this study investigated the effects of parent–child relationships and parental control on adolescent mental health issues. First, the study results indicated a significant negative correlation between parent–child relationships and adolescent mental health status: better parent–child relationships corresponded to lower levels of adolescent mental health issues. This finding aligned with previous research conclusions (Hazel et al., 2014). The previous study revealed a significant decline in the quality of parent–child relationships between ages 11–14 (McGue et al., 2005). Against the backdrop of the significant inhibitory effect of parent–child relationships on adolescent mental health, there should be an emphasis on enhancing parental awareness of fostering intimacy in parent–child relationships to prevent adolescent mental health issues.

Second, this study also noted that there was no significant direct correlation between parental control and adolescent mental health, which contradicted conclusions suggesting that parental control led to adolescent depression or promoted adolescent mental health (Darling et al., 2008; Leto et al., 2019). The possible reason was that parental control itself did not directly affect adolescent mental health but rather exerted its effects through moderating other variables.

Third, this study further found that parental control indeed moderated the relationship between parent–child relationships and adolescent mental health, indicating that high levels of parental control promoted the inhibitory effect of good parent–child relationships on adolescent mental issues. The moderating effect of parental control on parent–child relationships might be explained from a cultural diversity perspective. Baumrind suggested that the influence of parental discipline practiced on children was moderated by cultural backgrounds, with different cultures interpreting discipline differently (Baumrind, 1996). The concept of authoritarian parenting originated from American culture and exhibited controlling characteristics, whereas in Chinese culture, although parental control levels might be higher, “guan” in Chinese parenting culture, understood to denote parental responsibilities, embodied positive localized meanings and had surpassed Western parenting ideologies (Chao, 1994). Therefore, exploring the impact of parental control without considering social and cultural backgrounds might not be appropriate. Different motivations behind parental control in different cultures influenced perceptions, with collectivist countries emphasizing harmonious family relationships, which could lead to positive outcomes through parental control, thereby promoting adolescent mental health development (Dwairy & Achoui, 2010). Adolescents’ perception of parental motivations for discipline enhanced their acceptance of parental authority legitimacy, thereby alleviating parent–child conflicts caused by discipline. In conclusion, Asian cultures emphasized interpersonal relationships and dependence, and parental control may not necessarily threaten the self-esteem of children. On the contrary, parental supervision and management of children’s behavior were effective ways for parents to become closer to children and build good parent–child relationships, which was beneficial to adolescent mental health development.

The ultimate goal of parental discipline was to cultivate adolescents with self-reliance, achievement motivation, prosocial behavior, and social confidence (Steinberg, 2001). Perhaps because authoritarian discipline in the Western context was related to collectivism, norms of conformity, emotional restraint, and humility (Y. Xu et al., 2005), parental adherence to these traditional Chinese values was often seen as controlling children, which might hinder adolescent development. However, this understanding did not grasp the characteristics of Chinese parental discipline, as strict parental discipline demonstrated a positive value orientation of “hoping children can become dragons and daughters phoenixes”, aiming to cultivate children with outstanding qualities and abilities. Parents who perceived themselves as providing autonomy to their children and reducing control over their behavior often appeared indifferent and lacking in care, which reduced their children’s active disclosure of information to parents, leading to estrangement in parent–child relationships (Sorkhabi & Middaugh, 2014). Adolescents may even deliberately exhibit problematic behavior to express anger at their parents’ indifference, intending to attract parental attention and rebuild their security needs (Caffery & Erdman, 2000). Clearly, parental control contributed to the expression of parent–child relationships, thereby promoting the development of adolescent mental health, which was beneficial and necessary for both parents and adolescents.

However, this study also showed that parental control exerted a reverse moderating effect when parent–child relationships were poor. A study of Chinese–American families believed that parental demands for children in academics and family were “tiger parenting”, which led to academic stress, symptoms of depression, and more distant parent–child relationships in Chinese–American adolescents, without bringing good emotional adaptation ability (Kim et al., 2013). Supportive parent–child relationships meant perceiving warmth from parents, while high levels of parental control devoid of emotional support could lead to intense parent–child conflicts (Bi et al., 2018), which was detrimental to cultivating parent–child relationships and further led to adolescent mental health issues. These results suggested that parental control exerted a threshold effect on the moderating relationship between parent–child relationships and mental health. High levels of parental control amplified the positive impact of strong parent–child relationships, whereas low levels diminished it.

While we have explored the relationship between parent–child relationships, parental control, and adolescent mental health, it was crucial to consider the influence of different cultural contexts. These contexts could shape parenting practices and parent–child interactions in unique ways. When there was a mismatch between the parents’ cultural background and the dominant culture of their residence, parenting styles might diverge from local norms, potentially leading to strained family relationships. Furthermore, cultural factors could exert a multifaceted influence on parental control, affecting parental expectations, values, and interaction styles with children. Understanding these cultural nuances was essential for comprehending parent–child dynamics and providing tailored support to adolescents.

Finally, heterogeneous analysis results showed that high levels of parental control significantly positively moderated the inhibitory effect of parent–child relationships on mental health in adolescent girls. Surprisingly, this significant effect disappeared in adolescent boys. The possible reason was that on the one hand, the connection between parent–child relationships and depression was more significant for girls than for boys, especially father–daughter relationships (Mak et al., 2021); on the other hand, compared to boys, girls perceive higher levels of maternal behavioral control, and in adolescent girls, parental discipline behavior significantly predicted parent–child conflicts, a relationship that did not exist in adolescent boys (Shek, 2002, 2007). Therefore, girls were more susceptible to the influence of parental control and parent–child relationships. When parent–child relationships were poor, high levels of parental control might intensify parent–child conflicts, leading to mental health problems. This meant that parents needed to be cautious when dealing with their children during adolescence, and even more so with daughters.

There were some limitations to this study. Due to the cross-sectional design, this study could not establish causal relationships between variables. A longitudinal study would be better suited for this purpose. Additionally, the lack of data on parental control types limited the analysis. Future research should adopt longitudinal designs and explore the differential effects of psychological and behavioral control on adolescent mental health. While our study revealed an unexpected negative correlation between paternal education and adolescent mental health, further investigation is needed to understand the underlying mechanisms.

### 4.2. Conclusions

This study used nationally representative survey data and employed the OLS method to delve into the relationship between parent–child relationships, parental control, and adolescent mental health. The study demonstrated that good parent–child relationships significantly reduced adolescent mental health issues, while there was no significant direct correlation between parental control and adolescent mental health. However, parental control could moderate the impact of the parent–child relationship on adolescent mental health. High levels of parental control significantly positively promoted the inhibitory effect of good parent–child relationships on mental health issues, which was especially pronounced in adolescent girls. Moreover, the moderating effect of behavioral control exhibited a threshold effect, showing a positive moderating effect when parent–child relationships were above a certain level and a negative moderating effect when parent–child relationships were below this level.

This study confirmed the significant impact of parent–child relationships on adolescent mental health and, additionally, revealed the moderating role of parental control. The study suggested that the controversy surrounding the relationship between parental control and adolescent mental health may stem from solely discussing the independent impact of parental control without considering the context of parent–child relationships. Parental control itself may not directly affect adolescent mental health but rather functions through moderating the influence of parent–child relationships on mental health. Additionally, this study found gender differences in the moderating effect of parental control, providing a new perspective for understanding the relationship between parental control and adolescent mental health.

## Figures and Tables

**Table 1:** Descriptive analysis.

Variables	N	Average	Std Deviation	Mental Health	
				Coef	Sig
**Dependent Variable**					
Mental health	8962	10.85	4.19		
**Independent variables**					
Parent–child relationship	9124	2.60	0.48	−0.243	*p* < 0.001
**Moderating Variable**					
Parent control	9047	18.41	3.19	−0.055	*p* < 0.001
**Control variables**					
Age	9037	14.94	0.89	0.024	*p* < 0.05
Family economic status	8967	2.96	0.54	−0.100	*p* < 0.001
Mother’s education	9080	9.38	3.57	−0.057	*p* < 0.001
Father’s education	9057	10.20	3.13	−0.021	*p* < 0.05
Sex					*p* < 0.01
Male	4418	10.717			
Female	4476	10.986			
Household registration					*p* > 0.1
Rural	3899	10.924			
Urban	4828	10.789			

**Table 2:** Regression results.

Variables	Model 1	Model 2	Model 3	Model 4	Model 5
	Mental Health	Mental Health	Mental Health	Male	Female
Sex (male = 1)	−0.344 ***	−0.357 ***	−0.362 ***		
	(0.09)	(0.09)	(0.09)		
Age	0.064	0.063	0.068	0.114	0.017
	(0.05)	(0.05)	(0.05)	(0.08)	(0.07)
Family economic status	−0.635 ***	−0.635 ***	−0.631 ***	−0.751 ***	−0.475 ***
	(0.09)	(0.09)	(0.09)	(0.12)	(0.13)
Household Registration	0.333 **	0.345 ***	0.344 ***	0.414 **	0.283 *
(urban = 1)	(0.10)	(0.10)	(0.10)	(0.16)	(0.14)
Mother’s education	−0.062 ***	−0.065 ***	−0.064 ***	−0.036	−0.095 ***
	(0.02)	(0.02)	(0.02)	(0.03)	(0.02)
Father’s education	0.050 *	0.050 *	0.048 *	0.046	0.050 ^#^
	(0.02)	(0.02)	(0.02)	(0.03)	(0.03)
Parent–child relationship (PCR)	−2.074 ***	−2.069 ***	−2.110 ***	−2.114 ***	−2.090 ***
	(0.09)	(0.10)	(0.10)	(0.14)	(0.13)
Parent control (PC)		−0.017	−0.019	−0.039 ^#^	0.000
		(0.01)	(0.01)	(0.02)	(0.02)
PCR × PC			−0.087 **	−0.046	−0.138 ***
			(0.03)	(0.04)	(0.04)
_cons	17.258 ***	17.619 ***	17.722 ***	17.114 ***	17.921 ***
	(0.89)	(0.94)	(0.94)	(1.39)	(1.28)
*N*	8145	8041	8041	3941	4100
*R* ^2^	0.070	0.071	0.072	0.072	0.073
adj. *R*^2^	0.069	0.070	0.071	0.070	0.071

^#^ *p* < 0.1, * *p* < 0.05, ** *p* < 0.01, *** *p* < 0.001.

**Table 3:** Robustness test—control over time watching TV and returning home.

Variables	Model 6-1	Model 6-2	Model 6-3	Model 6-4
Sex (male = 1)	−0.352 ***	−0.357 ***	−0.357 ***	−0.359 ***
	(0.09)	(0.09)	(0.09)	(0.09)
Age	0.056	0.061	0.058	0.060
	(0.05)	(0.05)	(0.05)	(0.05)
Family’s economic condition	−0.643 ***	−0.640 ***	−0.634 ***	−0.631 ***
	(0.09)	(0.09)	(0.09)	(0.09)
Household registration	0.336 **	0.338 **	0.343 ***	0.341 ***
(urban = 1)	(0.10)	(0.10)	(0.10)	(0.10)
Mother’s education	−0.061 ***	−0.061 ***	−0.064 ***	−0.064 ***
	(0.02)	(0.02)	(0.02)	(0.02)
Father’s education	0.050 *	0.050 *	0.051 **	0.050 *
	(0.02)	(0.02)	(0.02)	(0.02)
Parent–child relationship (PCR)	−2.060 ***	−2.078 ***	−2.072 ***	−2.087 ***
	(0.09)	(0.09)	(0.09)	(0.09)
Control over the time on watching TV (CW)	−0.092	−0.104		
	(0.07)	(0.07)		
PCR × CW		−0.391 **		
		(0.14)		
Control over return home time (CRH)			−0.062	−0.073
			(0.07)	(0.07)
PCR × CRH				−0.365 **
				(0.14)
_cons	17.567 ***	17.579 ***	17.499 ***	17.554 ***
	(0.91)	(0.91)	(0.91)	(0.91)
*N*	8138	8138	8119	8119
*R* ^2^	0.070	0.071	0.070	0.071
adj. *R*^2^	0.069	0.070	0.069	0.070

* *p* < 0.05, ** *p* < 0.01, *** *p* < 0.001.

## Data Availability

The data presented in this study are available at http://ceps.ruc.edu.cn/.

## Supplementary Materials

The following supporting information can be downloaded at https://www.mdpi.com/article/10.3390/bs15010052/s1: Supplementary S1: Simple slope *t*-test.
