# Supplementary material for: Parent–Child Relationships, Parental Control, and Adolescent Mental Health: An Empirical Study Based on CEPS 2013–2014 Survey Data
Source: Behav Sci (Basel). 2025 Jan 8;15(1):52. doi: 10.3390/bs15010052 (PMC11761946; doi:10.3390/bs15010052)
Supplement: Supplementary file 1 [file behavsci-15-00052-s001.zip › behavsci-3353840-supplementary.pdf]

Simple slope T-test

Mental health issues	Coefficient	Std. err.	t	P>t	[95% conf.	interval]
	-0.0873777	0.0272046	-3.21	0.001	-0.1407057	-0.0340497
